# Advancements in the study of acute lung injury resulting from intestinal ischemia/reperfusion

**DOI:** 10.3389/fmed.2024.1399744

**Published:** 2024-06-12

**Authors:** Shihua Lv, Xudong Zhao, Can Ma, Dengming Zhao, Tian Sun, Wenchao Fu, Yuting Wei, Wenzhi Li

**Affiliations:** ^1^Key Laboratory of Anesthesia and Intensive Care Research, Harbin, China; ^2^Department of Anesthesiology, The Second Affiliated Hospital of Harbin Medical University, Harbin, China; ^3^Department of Hepatopancreatobiliary, The Second Affiliated Hospital of Harbin Medical University, Harbin, China

**Keywords:** intestinal ischemia/reperfusion, acute lung injury, animal models and evaluation indicators, pathophysiological mechanism, treatment strategy

## Abstract

Intestinal ischemia/reperfusion is a prevalent pathological process that can result in intestinal dysfunction, bacterial translocation, energy metabolism disturbances, and subsequent harm to distal tissues and organs via the circulatory system. Acute lung injury frequently arises as a complication of intestinal ischemia/reperfusion, exhibiting early onset and a grim prognosis. Without appropriate preventative measures and efficacious interventions, this condition may progress to acute respiratory distress syndrome and elevate mortality rates. Nonetheless, the precise mechanisms and efficacious treatments remain elusive. This paper synthesizes recent research models and pertinent injury evaluation criteria within the realm of acute lung injury induced by intestinal ischemia/reperfusion. The objective is to investigate the roles of pathophysiological mechanisms like oxidative stress, inflammatory response, apoptosis, ferroptosis, and pyroptosis; and to assess the strengths and limitations of current therapeutic approaches for acute lung injury stemming from intestinal ischemia/reperfusion. The goal is to elucidate potential targets for enhancing recovery rates, identify suitable treatment modalities, and offer insights for translating fundamental research into clinical applications.

## 1 Introduction

Intestinal ischemia/reperfusion (II/R) commonly occurs in the context of severe shock, infection, traumatic injury, mesenteric artery embolism, intestinal volvulus, intestinal obstruction, small bowel transplantation, liver transplantation, cardiopulmonary bypass surgery, and abdominal aortic aneurysm surgery. The associated morbidity and mortality remain significant (1). This is closely linked to the anatomical structure of the intestine and the body’s prioritization of ensuring blood supply to vital organs such as the heart and brain during acute ischemic stress (2). Following the relief of the cause of intestinal ischemia through surgery or other means, although the intestinal tissue can restore blood perfusion, the body undergoes a series of pathophysiological changes during II/R. These changes include ischemic injury due to hypoxia and lack of essential energy substances, resulting in damage to the intestinal tissue and increased vascular permeability. Subsequent reperfusion injury leads to metabolic disorders, activation of inflammatory signaling pathways and oxidative stress pathways, intracellular calcium overload, mitochondrial damage, and cell death (3). Oxygen and nitrogen free radicals, as well as inflammatory factors produced during II/R, are released into the blood, rapidly activating immune cells and triggering systemic inflammatory response syndrome (SIRS) (4). Additionally, the integrity of the intestinal mucosal barrier is compromised, allowing intestinal bacteria, endotoxin, and other substances to enter the circulatory system, causing distal tissue and organ damage, and potentially leading to multiple organ dysfunction syndrome (MODS) (5), posing a threat to patients’ lives.

**Figure 1:**
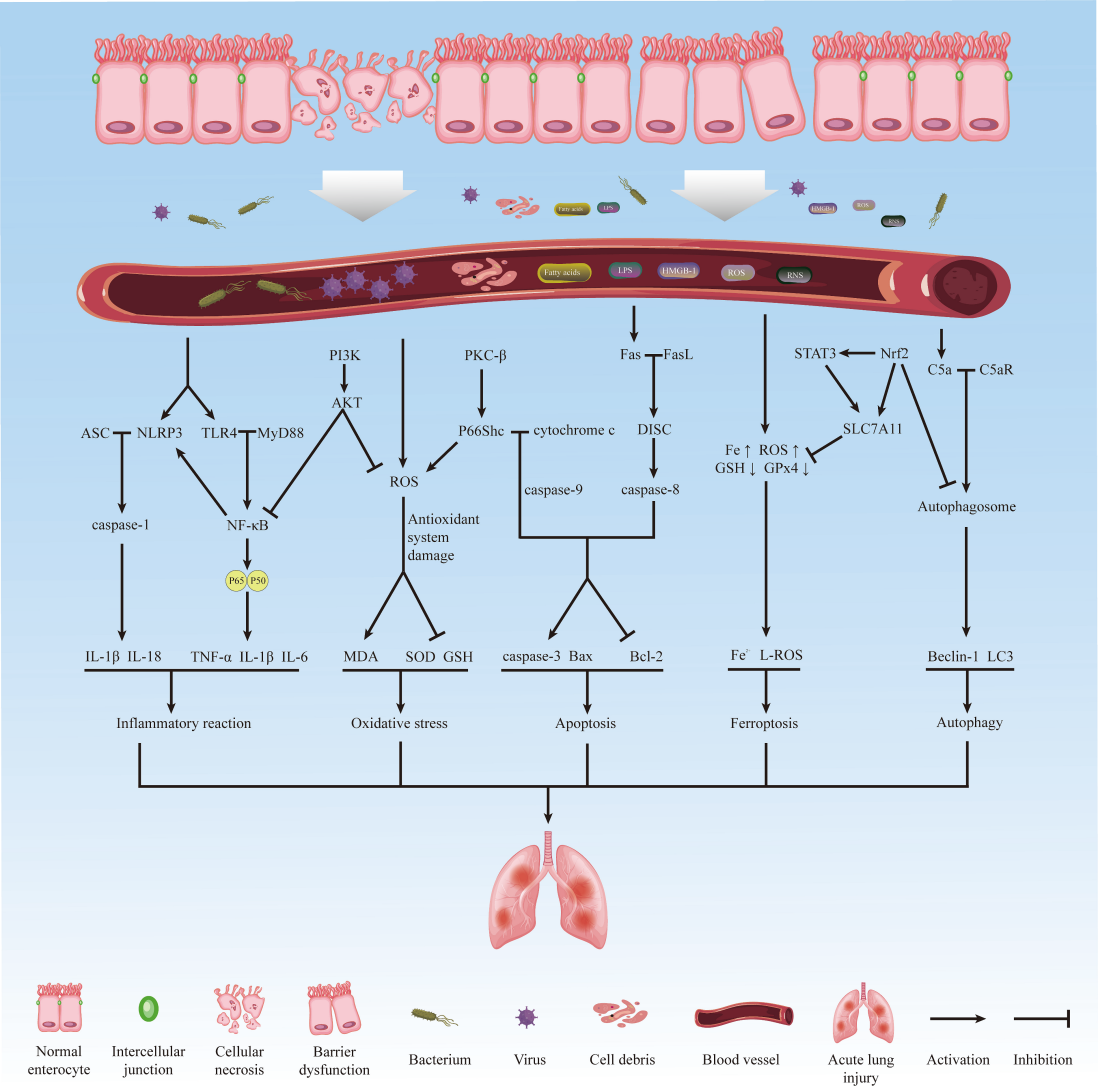
The main process and related mechanisms of acute lung injury induced by intestinal ischemia/reperfusion.

Among the various distal organs, the lung, being the sole recipient of all cardiac output, is highly sensitive to II/R and is particularly vulnerable. It exhibits early and severe characteristics of injury (6). Pathological manifestations of lung injury include alveolar epithelial and pulmonary capillary endothelial destruction, alveolar edema, neutrophil infiltration, and hyaline membrane formation (7), involving various interrelated pathophysiological mechanisms such as inflammatory response, oxidative stress, apoptosis, autophagy, and ferroptosis. Figure 1 summarizes the main processes and related mechanisms of acute lung injury induced by intestinal ischemia/reperfusion (Figure 1). Failure to adequately manage patients with acute lung injury caused by intestinal ischemia/reperfusion may lead to disease progression and subsequent development of acute respiratory distress syndrome (ARDS), characterized by a significantly high mortality rate of approximately 40% (8). However, the underlying mechanism of II/R-induced ALI (II/R-ALI) and effective methods to alleviate lung injury after II/R are not well-defined.

This article seeks to examine the animal models utilized in recent preclinical investigations of II/R-ALI, focusing on diagnostic criteria for acute lung injury, signal transduction pathways, injury-associated cytokines, treatment modalities, and existing challenges within this domain. The objective is to elucidate potential pathogenic mechanisms, identify relevant biomarkers, investigate novel targeted therapeutic approaches, and offer insights for both fundamental research and clinical translation.

## 2 Animal models and indicators for lung injury assessment

### 2.1 Animal models

Numerous studies on II/R-ALI lack standardized animal models and diagnostic criteria for lung injury. Researchers have employed various animal species to establish II/R models in order to fulfil the requirements for detecting corresponding indicators. For example, Li et al. (9) utilized C57BL/6J mice, Chen et al. (10) employed Sprague-Dawley rats, Bian et al. (11) utilized pigs, and Anderson et al. (12) utilized horses. Moreover, the timing of sample collection for intestinal tissue ischemia and reperfusion varies. The duration of intestinal ischemia induced by clamping the superior mesenteric artery ranged from 30 min to 2 h. The shortest duration for removing the artery clip to restore blood perfusion of the superior mesenteric artery was 60 min, while the longest was seven days.

### 2.2 Assessment of lung injury

In animal studies, the assessment of II/R-ALI primarily encompasses the following dimensions. Physiological impairment, such as reduced arterial partial pressure of oxygen (PaO_2_) in blood gas analysis; diminished lung compliance: decreased static compliance of lung tissue, elevated airway resistance; heightened permeability of the alveolar capillary membrane: increased total protein concentration in alveolar lavage fluid, augmented extravasation of FITC fluorescent dye, elevated extravascular lung water content, and increased wet/dry weight ratio of lung tissue; escalated pathological damage score of lung tissue: accumulation of neutrophils in alveoli or pulmonary interstitium; formation of hyaline membrane, presence of protein fragments in the alveolar space, thickening of alveolar walls; inflammatory response in lung tissue: increased absolute number of neutrophils in bronchoalveolar lavage fluid, heightened activity of myeloperoxidase (MPO) in lung tissue, and increased concentrations of pro-inflammatory cytokines (IL-1β, TNF-α, IL-6, IL-18) and chemokines (CXCL1, CXCL2, CXCL8) in lung tissue or bronchoalveolar lavage fluid (13); exacerbated oxidative stress: elevated concentrations of malondialdehyde (MDA) and reactive oxygen species (ROS) in lung tissue and blood, and decreased concentration of glutathione (GSH) and superoxide dismutase (SOD); apoptosis of lung tissue: increased number of TUNEL-positive cells in lung tissue, decreased ratio of Bcl-2/Bax, and increased expression of pro-apoptotic factors caspase-3, caspase-8, and caspase-9. Table 1 summarized recent animal models of II/R-induced lung injury and associated assessment indicators (Table 1).

**Table 1:** Animal models and lung injury assessment indicators for II/R-ALI.

Species	II/R model		Assessment of lung injury	References
	Ischemia time	Reperfusion time		
Mice	30 min	4 h	HE, MPO, MDA, FITC, occludin	(14)
	30 min	6 h	HE, water weight of lung, TNF-α, IL-6, IL-1β, MPO, IL-8, IL-18	(15)
	40 min	7 days	HE, BALF, TPC, TNF-α, IL-6, IL-1β, MPO, TUNEL	(16)
	45 min	2 h	HE, SOD, MPO, MDA, GSH, TNF-α, IL-1β, lung water, LDH, NLRP3, Bax, Bcl-2, caspase-3, CXCL-1	(17) (18)
	45 min	3 h	HE, TEM, water weight of lung, MDA	(19)
	45 min	2 h/24 h	MPO, EPO, iNOS, IL-6, IL-10, W/D, neutrophil and eosinophil number in lung tissue, SaO_2_	(20)
	45 min	5 h	MDA, MPO, water weight of lung	(21)
	1 h	1 h	HE, BALF, TPC, water weight of lung, GSH, MDA, HMGB1, TNF-α, IL-6, PaO_2_, HE, Masson, IL-1β	(7, 22)
	1 h	3 h	HE, MPO, caspase-3, TUNEL, TNF-α, IL-6	(23)
	1 h	4 h/20 h	HE, LDH, TUNEL, IL-6, MPO	(24)
	1 h	2 h/4 h/6 h/12 h	HE, NETs, HMGB1, CXCL1, CXCL2, TLR4, BALF, MPO	(6)
	90 min	30 min/1 h/90 min	HE, Masson, BALF, TPC, GSH, MDA, PaO_2_, water weight of lung, TUNEL, TLR-4, TEM, TNF-α, IL-6, Bax, Blc-2	(9, 25)
Rats	30 min	1 h/4 h/24 h	HE, MPO, MCs, neutrophil	(26)
	30 min	2 h	HE, NF-κB, MDA, iNOS, NOx, p65, TNF-α, IL-6, MPO, ICAM-1, Bcl-2	(27)
	40 min	8 h/16 h/24 h	HE, TNF-α, IL-10	(28)
	45 min	2 h	Pulmonary artery reactivity, circulating leukocytes number, ROS, SOD, IL-6, NF-κB, eNOS, IL-1β	(29)
	1 h	2 h	Caspase-1, mTOR, p70S6K, p65, NLRP3, TLR4, W/D, MPO, MDA, SOD, TNF-α, IL-6	(10, 30)
Pig	2 h	4 h	HE, MDA, SOD, MPO, W/D, Cst, Raw, PaO_2_, D_A-a_O_2_, PPI, BALF	(11)
Horse	2 h	2 h/6 h/12 h/18 h	HE, RR, total WBC count, total neutrophil count, IL-β, TNF-α, IL-10, TGF-β, caspase-3, caspase-8, caspase-9	(12)

HE, hematoxylin-eosin; MPO, myeloperoxidase; MDA, malondialdehyde; FITC, fluorescein isothiocyanate; BALF, bronchoalveolar lavage fluid; TPC, total protein concentration; TUNEL, terminal deoxynucleotidyl transferase-mediated dUTP nick-end Labeling; SOD, superoxide dismutase; GSH, glutathione; LDH, lactate dehydrogenase; TEM, transmission electron microscope; EPO, erythropoietin; NOS, nitric oxide synthase; W/D, wet/dry weight ratio; SaO_2_, arterial oxygen saturation; PaO_2_, arterial blood partial pressure of oxygen; NETs, neutrophil extracellular traps; MCs, mast cells; NO, nitrogen oxides; ROS, reactive oxygen species; Cst, static compliance; Raw, airway resistance; D_A-a_O_2_, alveolar-arterial oxygen pressure difference; PPI, pulmonary permeability index; RR, respiratory rate; WBC, white blood cell.

## 3 The mechanism of intestinal ischemia/reperfusion-induced acute lung injury

### 3.1 Oxidative stress

Oxidative stress is a pathological process characterized by excessive production of oxidizing substances and an imbalance in the body’s antioxidant defence system. This leads to the infiltration of inflammatory cells, increased secretion of proteases, and the generation of a large number of intermediate products, such as reactive oxygen species and active nitrogen, which the body is unable to neutralize. This process has detrimental effects on the body and is considered a crucial factor in ageing and disease (31). The classical mechanism of oxidative stress is crucial in II/R-ALI.

#### 3.1.1 Production of reactive oxygen species

ROS, primarily produced by mitochondria, are the metabolites of oxygen molecules and the highly reactive chemicals derived from them. They are direct participants and important biomarkers of oxidative stress (32). Under normal physiological conditions, cells produce a small amount of ROS as part of physiological processes such as pathogen inactivation, wound healing, and tissue repair (33). During intestinal ischemia, tissue cell metabolism slows down or stops, leading to the inability of mitochondrial metabolism to recover coenzymes (NADH, H^+^, and FADH_2_). Upon reperfusion, the release of a large number of electrons from the coenzymes exceeds the transfer capacity of the respiratory chain, producing a large amount of ROS. Additionally, the change in respiratory chain compounds caused by ROS exacerbates mitochondrial dysfunction and disrupts mitochondrial ROS production. Furthermore, nicotinamide adenine dinucleotide phosphate (NADPH) oxidase uses NADPH and H^+^ as substrates to catalyze the reduction of O_2_ to ROS (34). Calcium overload and reduced ATP synthesis lead to the activation of xanthine oxidase (XO) and the production of ROS. Nitric oxide synthase (NOS) exists as a homodimer under physiological conditions, while it exists as a monomer that contributes to the production of ROS during II/R, which is involved in producing ROS (35). A large amount of ROS produced by the body through the above pathways enters the blood circulation, accelerating ischemia/reperfusion injury. The excessive production of ROS overwhelms the body’s processing capacity of antioxidant substances, such as SOD, glutathione peroxidase (GSH-Px), and catalase (CAT), triggering systemic oxidative stress and tissue damage (36).

#### 3.1.2 Oxidative stress-induced lung injury

Studies have confirmed that oxidative stress can reduce the oligomerization of tight junctions (TJs) in the alveolar barrier, recruit neutrophils to cross the alveolar barrier, secrete cytotoxic substances, and increase pulmonary capillary permeability (37). Increased ROS production also leads to the decomposition of TJs in lung tissue, up-regulation of pro-inflammatory cytokines and chemokines, amplifying tissue damage and pulmonary edema, and accelerating the destruction of the alveolar barrier (38). In a model of II/R, the content of MDA in lung tissue increased. At the same time, the total antioxidant capacity (TAC) decreased, and the expression of inducible nitric oxide synthase (iNOS) and its product NOx in lung tissue increased by 5.5 times and 2.1 times, respectively. The total score of pathological injury of lung tissue was also increased significantly. This led to robust inflammatory reactions, alveolar edema, hemorrhage, and alveolar emphysema. However, treatment with the antioxidant cilostazol restored the balance between MDA and TAC, significantly alleviating lung injury. Furthermore, enhancing NADPH oxidase activity and mast cell activation were found to be related to II/R-ALI. Inhibition of mast cell degranulation can protect lung endothelial cells from oxidative stress and improve lung function (39).

#### 3.1.3 Oxidative stress-related signaling pathways

Studies have indicated that the PI3K/Akt signaling pathway is closely associated with oxidative stress, and its activation can effectively inhibit oxidative stress-induced apoptosis (40). PTEN, a negative regulator of the PI3K/Akt pathway, is enhanced in ischemia/reperfusion injury, leading to reduced Nrf2 activity, oxidative stress, and impaired intestinal and lung functions (41). Research has shown that bone marrow mesenchymal stem cell derived-exosomes (BMSC-exos) significantly regulate the PTEN/AKT/Nrf2 signaling pathway, reducing apoptosis and oxidative stress and improving intestinal injury (42). Additionally, miR-144-3p, which can inhibit the synthesis of target gene proteins, was found to target PTEN expression. Regulating BMSC-exos on the PTEN/AKT/Nrf2 pathway and oxidative stress is achieved by regulating miR-144-3p, providing evidence for the potential application of BMSC-exos in the treatment of ischemia/reperfusion injury (43).

### 3.2 Inflammatory response

As a crucial digestive organ, the intestine harbors a diverse microbial flora. During II/R, the release of intestinal flora into the bloodstream triggers SIRS (44). The activation of the NF-κB signaling pathway, Toll-like receptor 4 (TLR4) signaling pathway, and NOD-like receptor protein 3 (NLRP3) inflammasome are identified as the primary cause of ALI in this context.

#### 3.2.1 NF-κB signaling pathway

NF-κB is a rapid-acting nuclear transcription factor that governs essential cellular signal transduction pathways. Following I/R, the disruption of the intestinal mucosal barrier and compromised immune function led to the influx of bacteria, endotoxins, inflammatory mediators, and oxidative stress-related factors into the circulation. These substances bind to cell surface receptors and activate the IκB kinase (IKK) complex through various adaptors and signal kinases. The activation of IKK complex phosphorylates IκBα, an inhibitor of NF-κB, resulting in the release and translocation of NF-κB dimer to the nucleus. This process regulates the transcription of target genes, leading to the production and release of inflammatory mediators (45). Previous research has demonstrated that the activation of the NF-κB signaling pathway significantly upregulates the expression of inflammatory cytokines such as IL-1, IL-6, and TNF-α in lung tissue. This activation also increases neutrophil infiltration in lung tissue. It reduces the expression of intercellular adhesion molecule-1 (ICAM-1), resulting in lung tissue congestion, edema, and widespread infiltration of inflammatory cells in the alveoli and interstitial lung tissue. Additionally, it damages the integrity of the alveolar barrier (46). TNF-α, in particular, not only directly causes lung tissue damage but also forms a positive feedback loop with NF-κB, inducing endothelial cells to produce IL-1β, IL-6, and other inflammatory factors, thereby triggering a cascade effect of inflammatory factors and exacerbating secondary lung injury (15). In animal experiments, overexpression of miR-146 reduced the secretion of pro-inflammatory cytokines by inhibiting the TNF receptor-associated factor 6 (TRAF6)/NFκB signaling pathway, inhibited the apoptotic response in the intestine and lung tissues of mice with II/R-ALI, and protected mice from II/R-ALI (47).

#### 3.2.2 TLR4 signaling pathway

Toll-like receptors (TLRs) are a class of type I transmembrane protein receptors that identify pathogen-associated molecular patterns (PAMPs) on microorganisms and damage-associated molecular patterns (DAMPs) from host’s damaged cells and are crucial for innate immunity (48). TLR4 is a pattern recognition receptor within the TLRs family, primarily recognizing exogenous ligands such as lipopolysaccharide, as well as specific endogenous ligands including free fatty acids, heat shock proteins, and high mobility group protein B1 (HMGB1). Upon ligand binding, the TLR4 receptor domain interacts with cytoplasmic adaptor proteins (MyD88, TIRAP/MAL, and TRIF) to activate various signal transduction pathways, including extracellular signal-regulated kinase (ERK), c-Jun NH2 terminal kinase (JNK), mitogen-activated protein kinase (MAPK), and nuclear factor kappa B (NF-κB) pathways, resulting in the production of pro-inflammatory cytokines and chemokines (49). During II/R, intestinal wall permeability decreases, leading to increased circulating blood lipopolysaccharide levels. This triggers the TLR4 signal transduction pathway, inducing a cascade reaction through the myeloid differentiation factor 88-dependent factor, resulting in excessive activation of the mononuclear macrophage system, immune response, cytokine storm, and ultimately ALI (50).

HMGB1, a primary DAMP released by necrotic intestinal cells following II/R, activates the TLR4/MyD88 signaling pathway, driving neutrophil recruitment to lung tissue. Activated neutrophils can release DNA and granular proteins, forming cytotoxic neutrophil extracellular traps (NETs), which damage the alveolar barrier and exacerbate lung tissue injury. Blocking HMGB1 reverses these effects, protecting lung tissue from inflammation (6). In a porcine II/R model, propofol pretreatment decreased inflammatory factor and oxidative stress-related substance levels in lung tissue, improved II/R-induced hypoxemia and acidemia, reduced airway resistance, and protected lung function by inhibiting the expression of the HMGB1/TLR4/PKR signaling pathway (11). In the oxygen and glucose deprivation/reoxygenation (OGD/R) model of mouse lung epithelial cells, silencing Nrf2 increased OGD/R-induced upregulation of TLR4 and MyD88, and exacerbated apoptosis and autophagy, revealing the role of the Nrf2/TLR4/MyD88 axis in inflammation-related lung injury (25). The TLR4/NF-κB signaling pathway is a classical mechanism triggering an inflammatory response in the ischemia/reperfusion model. Activation of TLR4 can promote NF-κB p65 activation, stimulate downstream inflammatory factor and chemokine production, and lead to ALI. BMSC-exos can inhibit TLR4/NF-κB signal transduction, control the inflammatory response, down-regulate the critical protease caspase-3 in the apoptotic cascade, reduce pulmonary microvascular epithelial cell apoptosis, and play a protective role in lung function (51).

#### 3.2.3 NLRP3 inflammasome

NLRP3 is a receptor that contains NOD-, LRR-, and pyrin domain-containing 3. It has the ability to detect damage-associated molecular patterns (DAMPs) from pathogens and the host, thereby initiating the formation of NLRP3 inflammasome complex (52). As a major participant in II/R-ALI, NLRP3 becomes activated following II/R and aggregates with ASC (the adaptor protein in the NLRP3 inflammasome), which co-activate caspase-1 and promote the conversion of pro-IL-1β and pro-IL-18 into IL-1β and IL-18, causing acute inflammatory response and oxidative stress in lung tissue, leading to ALI (53). Furthermore, II/R stimulation can induce the production of intestinal lipid mediators, such as 12-HETE, 11-HETE, and 13-HODE, which have regulatory effects on both innate and adaptive immune responses. These mediators enhance the activation of NLRP3 inflammasome and the production of IL-1β in pulmonary vascular endothelial cells, contributing to ALI (54). It is now recognized that gut-derived lipid mediators hold the potential as regulators for treating and preventing II/R-related diseases. Over-activation of NLRP3 leads to the assembly of NLRP3 inflammasome complex and triggers pyroptosis through GSDMD-NT, resulting in plasma membrane rupture, production of inflammatory cytokines, and excessive release of LDH (55). Li et al. (17) observed increased expression levels of NLRP3 and NLRP3 inflammasome complex activation markers, such as caspase-1 and GSDMD, in *in vivo* mouse II/R models and cell experiments of OGD-R, along with elevated LDH activity.

### 3.3 Apoptosis

Apoptosis is a regulated cell death process characterized by cell shrinkage, plasma membrane protrusions, chromosome condensation, and nuclear fragmentation (56). Under normal physiological conditions, apoptosis occurs spontaneously in intestinal epithelial cells (IEC), maintaining the normal morphology and function of the intestine and ensuring intestinal homeostasis (57). However, under certain pathological conditions, apoptosis is the predominant outcome of inflammatory response and oxidative stress (58).

In the context of the II/R model, excessive apoptosis of IEC following ischemic stress leads to increased intestinal permeability, secondary bacterial translocation, and apoptosis of distal lung tissue through a series of complex pathophysiological reactions. The acute inflammatory response in lung tissue induced by II/R triggers the binding of the death receptor Fas to its ligand FasL in lung epithelial cells (59). Additionally, the tumor necrosis factor receptor-1 (TNFR1) binds to its ligands TNF-α and TNF-related apoptosis-inducing ligand (TRAIL), initiating the mechanism of exogenous apoptosis. This leads to the recruitment of adaptor proteins, such as the Fas-associated death domain (FADD), to form a death-inducing signaling complex (DISC) that activates caspase-8 and its downstream apoptotic proteins. Simultaneously, cytochrome c is released into the cytoplasm, triggering the activation of caspase-9-dependent caspase-3 and initiating the downstream apoptotic cascade (60). Furthermore, the expression ratio of Bcl-2/Bax protein in lung tissue decreases (17), and the number of positive cells increases significantly, as indicated by TUNEL staining (16). Previous studies have demonstrated that in the II/R-ALI model, TNF-α and reactive oxygen species (ROS) can initiate endogenous or exogenous apoptotic pathways, increase the formation of caspase-3 and promote cell death (61).

As a member of the Shc A family, p66Shc is expressed in most cells except hematopoietic cell lines and plays a crucial role in apoptosis by inducing DNA fragmentation, cytoskeleton degradation, and the formation of apoptotic bodies (62). Studies have shown that after II/R, protein kinase C-β (PKC-β) is activated explicitly in lung tissue (63), leading to a significant up-regulation of p66Shc expression and increased sensitivity of lung tissue to oxidative stress (64). The phosphorylation and activation of Ser36 of p66Shc by PKC-β result in its translocation to mitochondria, leading to mitochondrial dysfunction and promoting apoptosis of lung tissue cells (65). The specific inhibitor of PKC-βII, LY333531, has been found to significantly inhibit the activation of p66Shc, mitochondrial translocation, and binding to cytochrome c, thereby reducing II/R-induced acute lung tissue injury, inflammatory cell infiltration, oxidative stress, and apoptosis (66). Additionally, Feng et al. (64) found that the upstream regulator of the p66Shc pathway, prolyl-isomerase Pin1, plays a similar role to PKC-β. II/R increased Pin1 protein expression and enzyme activity, promoting the translocation of p66Shc to mitochondria and accelerating apoptosis. Injection of the Pin1 inhibitor Juglone before II/R in rats effectively reduced secondary lung injury and improved the survival rate after II/R in rats. Functional analysis showed that p66Shc silencing significantly reduced OGD/R-induced human colonic adenocarcinoma cell line Caco-2 damage, increased cell viability, reduced mitochondrial superoxide levels, and reduced TUNEL-positive cells and caspase-3 activation.

### 3.4 Ferroptosis

The concept of ferroptosis was initially introduced by Dixon et al. (67) in 2012 as a novel form of regulated cell death distinct from traditional cell death programs. Ferroptosis is characterized as an iron-dependent and caspase-independent non-apoptotic cell demise. The underlying mechanism involves iron catalyzing the generation of lipid free radicals and glutathione deficiency or inactivation of the lipid repair enzyme GSH peroxidase 4 (GPx4) (68), leading to impairment of the intracellular antioxidant system. This is manifested by the accumulation of iron and lipid reactive oxygen species (L-ROS) in mitochondria, reduction or disappearance of mitochondrial cristae, increased mitochondrial membrane density, and rupture of the mitochondrial outer membrane, ultimately resulting in cellular dysfunction (69). Previous research has demonstrated that polyunsaturated fatty acids, oxygen, iron, and antioxidants are pivotal factors in inducing ferroptosis (70, 71). Current investigations have established a close association between ferroptosis and various human diseases, including cardiovascular and cerebrovascular diseases, tumors, respiratory diseases, and ischemia/reperfusion injury (72).

Scholars have identified ferroptosis as a critical factor in II/R-ALI, and the inhibition of ferroptosis has been shown to have a protective effect against II/R-induced organ damage (9). In the II/R-ALI model, the levels of endogenous iron, ferrous iron, ferritin, and MDA in lung tissue were significantly elevated, while the level of reduced glutathione was markedly reduced (73). Transmission electron microscopy results revealed characteristic structural changes of ferroptosis in the mitochondria of type II alveolar epithelial cells in II/R mice (74). Conversely, intervention with ferrostatin-1 in mice with II/R-ALI reduced lung tissue damage, improved lung epithelial cell viability, and restored epithelial barrier function (75). These findings indicate that ferroptosis is a crucial participant in II/R-ALI.

Nuclear factor erythroid 2-related factor 2 (Nrf2) is a critical transcription factor that responds to intracellular oxidative stress and is closely associated with ferroptosis. It becomes activated in high oxidative stress conditions, leading to the transcription of target genes and binding to the antioxidant response element (ARE) in the nucleus. This activation promotes the translation of antioxidant and anti-inflammatory proteins (76), regulates the ferroptosis pathway by controlling glutathione, iron, and lipid metabolism, and influences mitochondrial function (77), ultimately playing a cytoprotective role. Studies have shown that Nrf2 activation in the nucleus can effectively prevent ALI (78). Additionally, the inhibitor of apoptosis-stimulating protein of p53 (iASPP) exerts p53-independent anti-ROS activity in the cytoplasm, promoting the accumulation and nuclear translocation of Nrf2, and increasing the expression of hypoxia-inducible factor-1α (Hif-1α). This process reduces the content of transferrin (TF) and ferritin-related proteins FTH1, NQO-1, and HO-1, thereby protecting cells from ferroptosis by regulating the Nrf2-dependent Nrf2/HIF-1/TF signaling pathway (9). Furthermore, Nrf2 silencing has been observed to exacerbate ferroptosis in lung epithelial cells and reduce the expression of telomerase reverse transcriptase (TERT) and SLC7A11. TERT has been reported to alleviate cell damage caused by ROS by going to mitochondria, while SLC7A11 is crucial in maintaining cell redox homeostasis (79, 80). The Nrf2/TERT/SLC7A11 axis has been shown to regulate ferroptosis, providing a potential new target for treating II/R-ALI (19). Additionally, signal transducer and activator of transcription 3 (STAT3) has been found to be involved in ferroptosis after II/R, being phosphorylated by Nrf2 to pSTAT3 and regulating SLC7A11 expression alongside Nrf2 (73). Further research is needed to explore the interaction between Nrf2 and STAT3 in ferroptosis. Activation of Nrf2 has also been shown to up-regulate the expression of heme oxygenase 1 (HO-1), another ROS detoxification enzyme (81). HO-1 content increases compensation with Nrf2 in II/R or OGD/R models, exerting an anti-iron ion effect (74). However, some studies have indicated that HO-1, in addition to acting as an antioxidant enzyme to prevent ferroptosis caused by oxidative stress after II/R, can induce ferroptosis under hypoxic conditions by releasing Fe^2+^ through its upstream regulator Hif-1α. Therefore, further investigation is required to understand the dual role of HO-1 in II/R-induced ferroptosis in lung tissue cells (22).

### 3.5 Autophagy

Autophagy is a ubiquitous biological process found in eukaryotic cells. It involves the formation of autophagosomes, which are double-layered membranes that encapsulate cytoplasmic proteins or organelles. These autophagosomes are derived from the rough endoplasmic reticulum and subsequently fuse with lysosomes to form autolysosomes. Within these autolysosomes, the enclosed contents are degraded, contributing to the regulation of homeostasis and immune response (82). Autophagy is considered a highly conserved cellular self-protection mechanism in evolution and is also recognized as a mechanism of cell death juxtaposed with apoptosis and necrosis (83). Its involvement in physiological and pathological processes makes its role in various diseases intricate and contradictory. In the context of II/R, an inflammation-related illness, the relationship between autophagy and disease progression is closely intertwined (84). Researchers have proposed differing perspectives on the role of autophagy in this context.

#### 3.5.1 Moderate autophagy

One potential mechanism is that following II/R, the body can effectively eliminate ectopic flora resulting from changes in intestinal mucosal permeability by activating autophagy. This process helps to maintain intestinal microecological balance, protect lung function, and regulate homeostasis (85, 86). For instance, using transmission electron microscopy, Yan et al. (25) observed a significant increase in the number of autophagosomes in lung tissue cells after II/R. Western blot results also confirmed the up-regulation of autophagosome markers LC3 and Beclin-1 in lung tissue. Further investigation into the mechanism revealed that Nrf2 could regulate the expression of p62, which is recruited into autophagosomes to participate in the degradation of target proteins through the autophagy-lysosomal pathway, thereby contributing to lung protection. Several studies have indicated that II/R can lead to autophagy dysfunction (87). Jiang et al. (88) also found autophagy dysfunction and abnormal activation of intestinal intraepithelial lymphocytes (IELs) in the II/R model. By promoting the expression of autophagy-related genes Beclin-1 and Atg16 and activating the NOD2/Beclin-1 pathway, the autophagy of IELs can be enhanced, the inflammatory response of intestinal mucosal epithelium can be reduced, and the energy balance and cell homeostasis of intestinal tissue during ischemia can be maintained, thereby improving ALI.

#### 3.5.2 Excessive autophagy

Other researchers have suggested that abnormal and excessive autophagy of intestinal cells after intestinal ischemia can exacerbate intestinal mucosal barrier dysfunction and secondary lung injury (89). Previous studies have shown that II/R-ALI can generate significant complement C5a in lung tissue (90). Alveolar macrophages, as resident macrophages in lung tissue, exhibit up-regulated expression of their C5a receptor (C5aR) (91). Upon C5a binding to C5aR in alveolar macrophages, downstream signal transduction is initiated, promoting autophagy and activating macrophage autophagy. This eventually leads to apoptosis of alveolar macrophages and disrupts lung homeostasis. To investigate the specific mechanism, Hu et al. (92) established atg5-deficient mice (Mf-ATG5/mice) in macrophages to inhibit the occurrence of macrophage autophagy. The results showed that the production of inflammatory cytokines in bronchoalveolar lavage fluid (BALF) of Mf-ATG5/mice was significantly reduced, the expression of autophagosome-related proteins was decreased, and lung injury was alleviated. It was confirmed that C5a interacted with C5aR to induce autophagy and subsequent apoptosis of alveolar macrophages by promoting the degradation of Bcl-2 by Beclin-1, which aggravated ALI. Conversely, inhibition of autophagy can alleviate II/R-ALI.

Therefore, the role of autophagy in II/R-ALI remains controversial, and a critical threshold may exist between its protective and promoting effects, necessitating further research exploration.

### 3.6 Others

Other than the previously mentioned classic mechanisms of II/R-ALI, scholars have suggested that pyroptosis, formation of NETs, and alterations in intestinal flora and their metabolites may also play a role.

#### 3.6.1 Pyroptosis

NLRP3-related pyroptosis has been shown to induce subsequent cell death by mediating the initial inflammatory response and plays an important role in various I/R injuries such as heart (93), liver (94), and kidney (95). Li et al. (17) found that in the intestine, like the above organs, when II/R leads to severe stress, NLRP3 inflammasome activates the caspase-1 pathway while causing an inflammatory response, promoting the maturation of IL-1β and GSDMD, and leads to pyroptosis of distal lung tissue cells. The same results were observed in the co-culture model of IEC-6 and MLE-12 cells induced by OGD/R. In the rat II/R model prepared by Yang et al. (96), immunofluorescence results showed that the expression level of pyroptosis marker GSDMD in lung tissue was significantly increased, and the application of inhibitors to block the formation of pyroptosis key factor NLRP3 complex can reverse the above phenomenon. It is suggested that pyroptosis is involved in the development of II/R-induced intestinal and distal lung injury, and inhibiting the occurrence of pyroptosis through appropriate pathways can effectively prevent organ damage.

#### 3.6.2 Neutrophil extracellular traps

Studies have found that after intestinal ischemia, HMGB1 released by necrotic intestinal epithelial cells drives lung recruitment and excessive activation of neutrophils through the MyD88 signaling pathway to undergo morphological changes, nuclear membrane rupture, the release of DNA and granular proteins into the cytoplasm, and finally plasma membrane rupture, leading to the formation of extracellular NETs, thereby aggravating ALI. Lung cell death and new NETs formation exacerbated II/R-ALI (97). Zhan et al. (6) also confirmed that NETs degradation and HMGB1 pathway inhibition can be used as an effective targeted therapy strategy to reduce ALI caused by intestinal ischemia-reperfusion, and may be suitable for critically ill patients with acute intestinal ischemic diseases. However, some scholars have also proposed that appropriate NETs formation can prevent bacterial translocation caused by II/R, promote the repair of intestinal mucosal injury, and maintain the stability of intestinal epithelium (98). Therefore, like other immunomodulatory methods, it is particularly important to balance the advantages and disadvantages of NETs formation in some specific cases. Future research should focus on the effects of NETs induction, inhibition, and degradation on pharmacological targets of diseases.

#### 3.6.3 Intestinal flora and their metabolites

In recent years, the rapid development of microbial sequencing technology has provided a good platform for researchers to explore the mechanism of disease occurrence and development from a more microscopic perspective. Through sequencing technology, scholars have found that II/R inevitably causes changes in the intestinal microenvironment and habitat microorganisms. This ecological imbalance is characterized by the proliferation of Enterobacteriaceae bacteria and the reduction of beneficial symbiotic bacteria (such as Lactobacillus) (99). This gut microbiota pattern can amplify the damage to organs such as the intestine, kidney, and lungs caused by II/R (100, 101). Studies have shown that succinic acid produced by gut microbiota metabolism acts as an important mediator of the gut-lung axis during II/R. 16S rRNA sequencing showed that II/R could lead to the imbalance of bacteria producing succinic acid and bacteria consuming succinic acid in the intestine. The Simpson index and the ratio of Firmicutes/Bacteroidetes in the lung microbiota decreased significantly, resulting in the production and accumulation of succinic acid in the lung through the gut-lung axis, followed by SUCNR1-dependent macrophage polarization and promotion of alveolar epithelial cell apoptosis to aggravate lung injury. The laboratory test results of patients with cardiopulmonary bypass also confirmed the above phenomenon, indicating that succinic acid may become a new biomarker for II/R-ALI (102).

From this point of view, the mechanisms of II/R-ALI are complex and closely related, such as the interaction between inflammation, oxidative stress, and pyroptosis, the interaction between NETs formation and gut microbiota (103), which still need more research in the future to reveal.

## 4 Treatment approach

### 4.1 Pharmacological intervention

Numerous domestic and international researchers have conducted extensive investigations into drug therapies targeting the pathophysiological mechanisms of II/R-ALI, yielding significant advancements. In addition to the well-established anti-inflammatory, antioxidant, and anti-apoptotic medications, surprising findings have emerged regarding the protective effects of propofol (104), dexmedetomidine (105), and other anesthetic agents commonly utilized during surgical procedures. It is worth mentioning that a clinical case report published in 2023 mentioned that the use of propofol during intestinal transplantation in a 20-year-old male infant effectively reduced the secondary damage caused by II/R (106). These anesthetic drugs not only contribute to patient comfort but also demonstrate advantages in safeguarding perioperative organ function. However, more prospective and large-scale clinical trials in the future are still needed to provide strong evidence to support the reliability of laboratory-proven methods for the safe and effective clinical application of patients diseases.

With the increasing public attention to their health in recent years, people gradually realize the irreplaceable role of intestinal flora and its metabolites in the occurrence and development of diseases. Microbiota transplantation, oral probiotics and the use of gut microbiota-derived metabolites have been proven to be prospective methods for the prevention and treatment of II/R-ALI, which can be safely and effectively applied in clinical practice (107, 108).

Moreover, drug administration has evolved from traditional oral or intravenous routes to the utilization of extracellular vesicles, such as exosomes (109), to facilitate targeted drug delivery to specific sites for pharmacological effects. At present, the separation of intestinal-derived exosomes in mouse II/R models can be realized at the technical level, which will be very helpful for researchers to conduct follow-up clinical trials (110).

### 4.2 Surgical intervention

Operative treatments encompass ischemic preconditioning (IPC) and remote ischemic preconditioning (RIPC).

IPC involves subjecting organs or tissues to one or more transient ischemia/reperfusion cycles before prolonged ischemia and reperfusion (111). Wang et al. (112) confirmed that intestinal IPC enhances the tolerance of the intestine and distal organs to subsequent prolonged ischemia, significantly enhances the body’s antioxidant capacity, suppresses the release of pro-inflammatory cytokines to mitigate SIRS, and reduces II/R-induced lung tissue apoptosis.

Remote ischemic preconditioning refers to the application of repeated transient non-lethal ischemia/reperfusion in organs or limbs to shield against subsequent distal organ ischemia/reperfusion injury. Hummitzsch et al. (113) demonstrated that subjecting Wistar rats to three bilateral hindlimb ischemic preconditioning sessions for 5 min each time significantly ameliorated local and distal intestinal damage caused by subsequent II/R. This may be due to the biologically active substances produced by the body after stimulation and released into the systemic circulation are transferred to the distal target organ or tissue for protection.

Although IPC has achieved good results in the prevention of II/R injury in animal experiments, it will bring side effects to patients in clinical practice. On the contrary, RIPC, an effective, simple and low-risk method, can be easily induced by transient blood flow obstruction using blood pressure cuffs (114), thereby enhancing its clinical translational potential. Other studies have corroborated that combined pretreatment with IPC and RIPC exerts a significantly more robust protective effect in the late stage of II/R (115). Future research can explore how to combine the two safely and effectively.

### 4.3 Stem cell therapy

Mesenchymal stem cells (MSCs) are pluripotent cells derived from the mesoderm, possessing self-renewal potential. As a prominent member of the stem cell family, MSCs find wide application in various clinical diseases (116). Research has demonstrated the protective effect of bone marrow-derived mesenchymal stem cells (BMSCs) on II/R-ALI (117). The mechanism primarily involves two categories: (1) exogenous MSCs migrate to locally damaged intestinal tissues and differentiate into IEC, thereby ensuring the integrity of the intestinal barrier, reducing bacterial translocation, and mitigating distal lung injury; (2) exogenous MSCs exert anti-inflammatory, anti-oxidative, anti-apoptotic effects, promote cell proliferation, and angiogenesis through the release of cytokines. However, the protective effect of MSCs on II/R injury is limited to basic experiments such as cells and animals, and there is no relevant clinical study. Scholars are trying to make more MSCs reach the target tissue after intravenous injection to play a role and improve the efficiency of treatment. MSCs offer valuable insights for future research and clinical trials, representing a promising new treatment approach for II/R injury.

### 4.4 Traditional Chinese medicine therapy

With its extensive historical background, traditional Chinese medicine has witnessed significant growth in treatment approaches as an understanding of its theory has evolved. Currently, prominent traditional Chinese medicine treatments for II/R-induced systemic diseases include conventional Chinese medicine treatment and acupuncture treatment. The effectiveness of Artesunate (18), Corilagin (17, 118), and Nervilifordin F (30), active ingredients of *Artemisia annua*, ellagic tannin, and *Nervilia fordii*, has been confirmed in preclinical studies of II/R-related diseases. The potential mechanism aligns with the aspects mentioned above. Traditional Chinese medicine offers the advantages of multi-target, multi-channel, multi-level, and minimal adverse reactions, providing a crucial guarantee for its safe and practical application in clinical practice. Acupuncture is a traditional Chinese medicine therapy. However, acupoint electrical stimulation (119) is a novel treatment method combining modern electroacupuncture therapy with standard acupuncture, which has the advantages of being non-invasive, safe, and not limited by time and place. Acupoint electrical stimulation has been clinically utilized for many years to prevent and treat diseases caused by ischemia/reperfusion of various organs and gastrointestinal diseases (120), yielding satisfactory results. It achieves the effect of “Fuzheng Quxie” and balancing “Yin and Yang” by stimulating the conduction of meridians and collaterals of the whole body and adjusting “Gas-Blood and Zang-Fu.” Geng et al. (121) confirmed that acupoint electrical stimulation therapy not only directly improves systemic inflammation caused by II/R but also indirectly promotes the differentiation of MSCs, playing a synergistic therapeutic role. Therefore, the combination of traditional Chinese and Western medicine holds broad application prospects, offering new hope for treating various II/R-induced diseases.

### 4.5 Others

**Figure 2:**
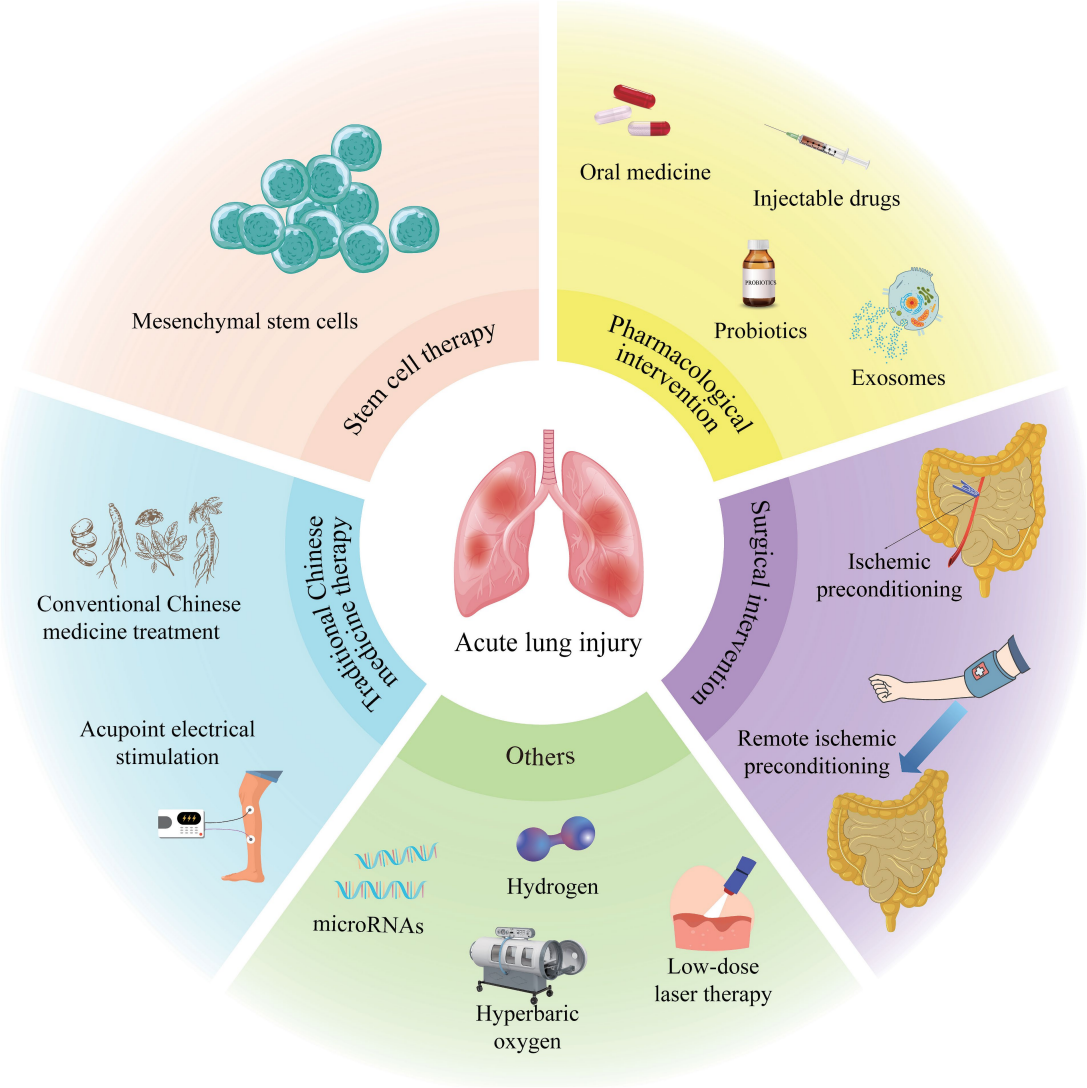
Treatment of acute lung injury induced by intestinal ischemia/reperfusion.

In the process of exploring the treatment of II/R-ALI, scientists have also proposed that low-dose laser therapy (122), hyperbaric oxygen therapy (123), hydrogen inhalation therapy (124), and microRNA-targeted regulation strategies (125) have specific mitigating effects on II/R-ALI (Figure 2). However, the specific dosage, treatment time and appropriate administration method still need to be further studied, which will increase the challenge of clinical transformation of treatment methods.

## 5 Summarized and prospected

In recent years, domestic and international scholars have conducted extensive research and exploration into the pathogenesis and treatment of II/R-ALI animal models. They have elucidated the mechanisms of the inflammatory response, oxidative stress, apoptosis, ferroptosis, autophagy, and changes in the intestinal flora and their metabolites caused by II/R-ALI, leading to pathological changes in distal lung tissue. Significant progress has been made in strategies aimed at improving or treating ALI. However, current research on II/R-ALI faces several challenges that hinder the advancement of disease treatment. Firstly, most studies about II/R-ALI are still in the preclinical research stage, primarily involving animal models or cell experiments, and there is a need for more relevant clinical case studies. Secondly, the pathophysiological and molecular mechanisms of II/R-ALI are complex, interrelated, and mutually reinforcing, with diverse manifestations of damage, making it difficult to intervene accurately against a single mechanism or target. This significantly complicates the exploration of disease treatment methods. Finally, the timing of administration, dosage, adverse reactions, pharmacokinetics, and toxicological effects of existing therapeutic drugs still need to be determined. The treatment efficiency is low, as only a few MSCs reach the target tissue after intravenous injection. The use strategy of acupoint electrical stimulation therapy in treating cerebral ischemia/reperfusion and myocardial ischemia/reperfusion injury is relatively clear. Still, the exact mechanism of the therapeutic effect of II/R-ALI remains to be explored. Although many methods for the treatment of II/R-ALI have their advantages, they still have some limitations, which makes it impossible for scholars to reach a consensus on this, which will increase the challenge of clinical transformation of existing treatment methods. Therefore, actively seeking vital pathogenic mechanisms, proposing new research perspectives and treatment approaches, accelerating the development of clinical trials, and early intervention to halt the progress of II/R-ALI are expected to improve the cure rate of II/R-ALI.

## Author contributions

SL: Writing – original draft, Methodology, Conceptualization. XZ: Writing – original draft, Visualization, Methodology. CM: Writing – original draft, Formal analysis. DZ: Writing – review & editing. TS: Writing – review & editing. WF: Writing – review & editing, Formal analysis. YW: Writing – review & editing. WL: Writing – review & editing, Supervision, Conceptualization.
